# Olive-Leaf Extracts Modulate Inflammation and Oxidative Stress Associated with Human *H. pylori* Infection

**DOI:** 10.3390/antiox10122030

**Published:** 2021-12-20

**Authors:** Jose Manuel Silvan, Esperanza Guerrero-Hurtado, Alba Gutiérrez-Docio, Teresa Alarcón-Cavero, Marin Prodanov, Adolfo J. Martinez-Rodriguez

**Affiliations:** 1 Microbiology and Food Biocatalysis Group (MICROBIO), Department of Biotechnology and Food Microbiology, Institute of Food Science Research (CIAL, CSIC-UAM), Cantoblanco Campus, Autonomous University of Madrid, C/Nicolás Cabrera 9, 28049 Madrid, Spain; 2 Department of Production and Characterization of Novel Foods, Institute of Food Science Research (CIAL, CEI, CSIC-UAM), Cantoblanco Campus, Autonomous University of Madrid, C/Nicolás Cabrera 9, 28049 Madrid, Spain; esperanza.guerrero@estudiante.uam.es (E.G.-H.); alba.gutierrez@uam.es (A.G.-D.); marin.prodanov@uam.es (M.P.); 3 Microbiology Department, Hospital Universitario de La Princesa, 28006 Madrid, Spain; talarcon@helicobacterspain.com; 4 Department of Preventive Medicine, Public Health and Microbiology, School of Medicine, Autonomous University of Madrid, 28029 Madrid, Spain

**Keywords:** olive-leaf extract, *Helicobacter pylori*, antioxidant activity, anti-inflammatory activity, HPLC-PAD-MS characterization, hydroxytyrosol, oleuropein, antibacterial activity

## Abstract

*Helicobacter pylori* (*H. pylori*) is one of the major human pathogens and the main cause of pathological damages that can progress from chronic gastritis to gastric cancer. During the colonization of gastric mucosa, this bacterium provokes a strong inflammatory response and subsequent oxidative process, which are associated with tissue damage. Therefore, the objective of this research was to evaluate the ability of two olive-leaf extracts (E1 and E2) to modulate the inflammatory response and oxidative stress in *H. pylori*-infected human gastric AGS cells. The obtained results showed that both extracts significantly decreased interleukin-8 (IL-8) secretion and reactive oxygen species (ROS) production in human gastric AGS cells. Both extracts also showed antibacterial activity against different *H. pylori* strains. HPLC-PAD-MS characterization demonstrated that extract E1 was mainly composed of highly hydrophilic compounds, such as hydroxytyrosol (HT) and its glucosides, and it was the most effective extract as an antibacterial agent. In contrast, extract E2 was composed mostly of moderately hydrophilic compounds, such as oleuropein (OLE), and it was more effective than extract E1 as an anti-inflammatory agent. Both extracts exhibited similar potential to decrease ROS production. These results show the importance of standardizing the extract composition according to the bioactive properties that should be potentiated.

## 1. Introduction

The growing of olive tree (*Olea europaea* L.) for production of olive oil and table olives is a widely established practice in the Mediterranean countries, where Spain is a main world producer. However, olive tree cultivation and olive oil processing generate large amounts of residues and byproducts, including olive mill wastewaters, olive pomaces, and olive leaves, which represents an important environmental problem when they are not processed correctly, due mainly to their high organic content and phytotoxicity [1,2]. However, these byproducts still contain high amounts of bioactive compounds that should be considered as potential low-cost sources of antioxidants and carbohydrates [1,3]. Therefore, valorization of these byproducts for their recovery and/or biotransformation is an alternative that could change the current treatment and disposal of these byproducts into products of high added value.

In this regard, the olive leaves (mixture of leaves and branches) are the largest byproduct obtained during olive tree pruning, and olive harvesting and cleaning [4]. The amount of olive leaves accumulated annually in this way may exceed 1 million tons [2].

The leaves of olive trees have been widely used as a folk medicine for many centuries. Nowadays, there is an increasing interest in the scientific community and in different industrial sectors (food supplements, cosmetic, and pharmaceutical industries) for their health benefits related to the presence of a huge diversity of secondary olive metabolites, such as phenolic and secoiridoid compounds [5,6]. The secondary metabolite composition varies depending on several factors, such as olive tree variety, growing, climatic and storage conditions, etc. Among them, the most abundant phenolic compounds are oleuropein (OLE) and hydroxytyrosol (HT) [7,8]. Other relevant compounds present in olive leaves are some secoiridoids (elenolic and demethyl elenolic acids and their glycosidic forms); flavones (luteolin, luteolin-7-glucoside, apigenin-7-glucoside, diosmetin, and diosmetin-7-glucoside); flavonols (rutin and kaempferol); flavan-3-ols (catechin), and phenolic acids (tyrosol, caffeic acid, chlorogenic acid, cinnamic acid, and vanillic acid) [1].

It is considered that olive leaves possess the highest antioxidant and anti-inflammatory activity amongst the different parts of the olive tree due to their high content in bioactive phenolic compounds [6,9]. These relevant properties may be due to their ability to chelate metal ions that catalyze free radical generation reactions [10], as well as their ability to inhibit many inflammatory enzymes, such as lipoxygenases [11]. Moreover, it has been observed that olive-leaf extracts are able to attenuate gastric and intestinal inflammation, and this positive effect has been associated with a modulation of the altered immune response due to its antioxidant capacity [12,13,14].

HT and OLE have been intensively studied for their contribution to the antioxidant and anti-inflammatory properties of olive-leaf extracts. HT has been associated with a strong antioxidant activity for its ability to act as a free-radical scavenger and metal-chelator, also increasing the endogenous defense systems against oxidative stress, by activating different cellular signaling pathways related to the attenuation of several proinflammatory interleukins [15,16]. OLE has also exhibited anti-inflammatory and antioxidant effects, both in vitro and in vivo, at low concentrations. These interesting pharmacological effects are mainly attributed to its ability to scavenge the reactive oxygen species (ROS) and to inhibit the proinflammatory neutrophil functions [9,17,18]. Moreover, antibacterial activity against Gram-positive and Gram-negative bacteria has been reported for olive leaves, and these results have been correlated with the presence of phenolic compounds, such as OLE and HT [19,20].

*Helicobacter pylori* (*H. pylori*) is formally recognized as a bacterial carcinogen [21], and is one of the most successful human pathogens, as over half of the world’s population is colonized with this Gram-negative bacterium [22]. *H. pylori* infection can cause various gastrointestinal diseases, ranging from chronic active gastritis without clinical symptoms to peptic ulceration, gastric adenocarcinoma, and gastric mucosa-associated lymphoid tissue lymphoma, and other extragastric pathologies [23]. Moreover, unless eradication therapy is implemented, the infection may persist for life. The chronic active gastritis is related to persistent colonization of the gastric mucosa by *H. pylori*, and most other severe gastric disorders are the consequence of a chronic mucosal inflammatory process mediated by activation of neutrophils and macrophages triggering the secretion of proinflammatory cytokines and chemokines, mainly interleukin-8 (IL-8) [24,25]. Activation of these inflammatory cells (neutrophils and macrophages) also provides an increase of ROS generation at the site of inflammation. This process results in cell dysfunction and tissue injury in the absence of antioxidants, and eventually can lead to oxidative DNA damage activating signaling pathways implicated in the pathogenesis of gastric carcinogenesis [26,27]. Normally, inflammation response and ROS production help in the clearance of bacterial infections [28], but if these processes are prolonged or inefficient, as occurs during *H. pylori* infection, both events are considered to possess special relevance in the progression to more severe tissue damages [29]. This chronic inflammation has been directly related to histopathological changes of the mucosa that can evolve from gastritis to gastric cancer, as was described by Correa et al. [30]. For this reason, a particular interest has been shown in the search for alternative treatments to antibiotics in *H. pylori* therapy. Although olive-leaf extracts are widely used due to their different bioactive properties [31], there are few studies concerning their efficacy against *H. pylori,* and they are mainly focused on the antibacterial properties, with contradictory results [20,32,33]. The use of olive-leaf extracts in the control of inflammation and oxidative stress associated with human *H. pylori* infection is largely unknown. This is despite the importance of both processes in the progression of the pathologies associated with *H. pylori* infection. In the present work, the anti-inflammatory, antioxidant, and antibacterial activities of two different olive-leaf extracts (E1 and E2) are studied against six different *H. pylori* strains. The chemical composition of the olive-leaf extracts are characterized, and relationship with bioactivities is discussed.

## 2. Materials and Methods

### 2.1. Source of Olive-Leaf Extracts, Reagents, and Pure Reference Substances Used in Their Chemical Characterization

Olive-leaf extracts (E1 and E2) were provided by Pharmactive Biotech Products S.L. (Madrid, Spain). Extract E1 was standardized in 4% elenolic acid and its derivates (Isenolic^®^) and extract E2 was standardized in 20% of OLE (Olivactive^®^).

High-performance liquid chromatography (HPLC)-grade water was obtained by a Milli-Q purification system from Millipore Corp. (Bedford, MA, USA). HPLC-grade acetonitrile was purchased from Merck (Dramstadt, Germany) and acetic acid (99.8%) from Labbox Labware S.L. (Madrid, Spain). HPLC-grade pure reference substances were acquired as follows: *trans*-4,5-DCQA (*trans*-4,5-dicaffeoylquinic acid) (>95%), quercetin (>95%), 4-HPE-EA-glucoside (ligustroside) (>96.2%), and 3,4-DHPE-EA-glucoside (oleuropein) (>98%) from Merck. Elenolic acid (EA) (>98%) and luteolin (>95%) were purchased from Toronto Research Chemicals (Toronto, ON, Canada), whereas 3,4-DHBA (protocatechuic acid) (>90%), 4-HPE (tyrosol) (>95%), *trans*-3,4-DHCA (*trans*-caffeic acid) (>99%), *trans*-4-HCA (*trans*-4-coumaric acid) (>98%), *trans*-3-M,4-HCA (*trans*-ferulic acid) (>98%), quercetin 3-*O*-rhamnoside (quercitrin) (>93.3%), luteolin 3′,7-di-*O*-glucoside (>97%), eriodictyol-7-*O*-rutinoside (>98%), eriodictyol 7-*O*-glucoside (>98%), luteolin 7-*O*-glucoside (>98%), and 3,4-DHPE caffeoyl glucoside (verbascoside) (>95%) were obtained from Extrasynthese (Genay, France). EA 2-glucoside (oleoside 11-methyl ester) (>98%), EMA 2-glucoside (secoxyloganin) (>99%), 3,4-DHPE (hydroxytyrosol) (>90%), quercetin 3-*O*-glucoside (isoquercitrin) (>99%), apigenin 7-*O*-glucuronide (>90%), and luteolin 4′-methyl ether 7-*O*-glucoside (diosmin) (>90%) were purchased from PhytoLab GmbH & Co. KG (Vestenbergsgreuth, Germany). Apigenin 6,8-di-C-glucoside (>95%) was obtained from Glentham Life Sciences (Corsham, UK) and apigenin 7-*O*-rutinoside (isorhoifolin) (>99.9%) was obtained from Biosynth AG (Switzerland). The 3,4-dihydroxyphenylglycol (3,4-DHPG) (75%) was provided by Prof. Juan Fernández-Bolaños from Instituto de la Grasa (CSIC) (Sevilla, Spain).

### 2.2. Chemical Characterization of Olive-Leaf Extracts

Solutions of 2, 10, and 20 mg/mL of extracts E1 and E2 were prepared in triplicate (*n* = 3) in water and methanol, respectively, and were analyzed by reverse-phase HPLC (RP-HPLC), coupled to photodiode array detector and mass spectrometry (MS) detector with electrospray ionization source (RP-HPLC-PAD-MS(ESI)) as described by Silvan et al. [34].

Samples of 3,4-DHBA, 3,4-DHPE, 4-HPE, 3,4-DHPE-EA-glucoside, 4-HPE-EA-glucoside, 3,4-DHPE caffeoyl glucoside, quercetin, quercetin 3-*O*-glucoside, quercetin 3-*O*-rhamnoside, apigenin 7-*O*-glucuronide, apigenin 6,8-di-C-glucoside, apigenin 7-*O*-rutinoside, luteolin, luteolin 3′,7-di-*O*-glucoside, luteolin 7-*O*-glucoside, luteolin 4′-*O*-methyl, 7-*O*-glucoside, eriodictyol 7-*O*-rutinoside, eriodictyol 7-*O*-glucoside, EA, EA 2-glucoside, EMA 2-glucoside, *trans*-3,4-DHCA, *trans*-4-HCA*, trans*-3-M,4-HCA, and *trans*-4,5-DCQA were identified unambiguously by coelution and comparison with their retention time, order of elution, UV spectra, and pseudomolecular and fragment ion masses of the corresponding pure reference substances, and quantified according to the calibration curves of each of them. The glucosides of 3,4-DHBA and 3,4-DHPE were identified tentatively by using their corresponding retention time, order of elution, UV spectra, pseudomolecular, diagnostic fragment ion masses, and bibliographic data [35,36,37]. Then, 3,4-DHBA glucoside was quantified as equivalents of 3,4-DHBA and the three 3,4-DHPE glucosides were quantified as equivalents of 3,4-DHPE. Results of quantification were expressed as mean value ± standard deviation (*n* = 3) on dry matter (mg/100 g).

### 2.3. H. pylori Strains, Growth Media, and Culture Conditions

Six strains of *H. pylori* (Hp44, Hp48, Hp53, Hp58, Hp59, and Hp61) were isolated in the Microbiology Department of Hospital La Princesa (Madrid, Spain) from gastric biopsies. Selective (Pylori agar) (BioMerieux, Madrid, Spain) and nonselective media (Blood-supplemented Columbia Agar) (BioMerieux) were used for culturing biopsies. *H. pylori* strains were stored at −80 °C in Brucella Broth (BB) (Becton, Dickinson, & Co., Madrid, Spain) plus 20% glycerol. Müeller-Hinton agar supplemented with 5% defibrinated sheep blood (MHB) (Becton, Dickinson, & Co, Franklin, NJ, USA) was used as agar-plating medium. BB supplemented with 10% horse serum (HS) (Biowest, Barcelona, Spain) was the liquid medium. The inoculum for *H. pylori* strains were prepared as follows: 200 μL of frozen strains were inoculated in a MHB plate and incubated in a variable atmosphere incubator (VAIN) (85% N2, 10% CO_2_, and 5% O_2_) (MACS-VA500, Don Whitley Scientific, Bingley, UK) at 37 °C for 72 h. Bacterial inoculum for the different assays was preparing suspended *H. pylori* colonies grown in a MHB plate in 2 mL of BB + 10% HS or culture medium cell (~1 × 10^8^ colony forming units (CFU/mL).

### 2.4. Human Gastric Epithelial Cell Culture Conditions

AGS cells (human gastric epithelial cell line) were obtained from the American Type Culture Collection (ATCC). Dulbecco’s Modified Eagle’s Medium/F12 (DMEM/F12) (Lonza, Madrid, Spain) supplemented with 10% fetal bovine serum (FBS) (Hyclone, GE Healthcare, Logan, UK) and 1% penicillin/streptomycin (5000 U/mL) (Lonza) was used to culture the cells. Around 1 × 10^6^ cells were plated in 75 cm^2^ culture flasks (Sarstedt, Barcelona, Spain) and maintained at 37 °C until 90% of cell confluence under 5% CO_2_ in a humidified incubator. Cell culture medium was changed every 2 days. Cell subculturing was carried out before a confluent monolayer appeared. Experiments were developed between passage 10 to passage 30 to ensure cell uniformity and reproducibility.

### 2.5. Evaluation of Cytotoxicity of Olive-Leaf Extracts

Cytotoxicity of olive-leaf extracts was evaluated previously to the antioxidant and anti-inflammatory assays. MTT (3,4,5-dimethylthiazol-2,5-diphenyl-tetrazolium bromide) (Sigma, Madrid, Spain) reduction assay was used to determine AGS cell viability [38]. Confluent cell cultures (~90%) were trypsinized (Trypsin/EDTA 170,000 U/L) (Lonza) and cells were seeded (~5 × 10^4^ cells per well) in 96-well plates (Sarstedt) and incubated in culture medium at 37 °C under 5% CO_2_ in a humidifier incubator for 24 h. Serum-free medium containing the extracts (at 2 mg/mL final concentration) was used to replace cell culture medium, and cells were incubated at 37 °C under 5% CO_2_ for 24 h. Non-treated cells (experimental control) were incubated in serum-free medium without extracts. Then, cells were washed with phosphate buffered saline (PBS) (Lonza), and medium was replaced by 200 μL of serum-free medium. After 1 h of incubation, 20 μL of an MTT solution in PBS (5 mg/mL) was added to each well for the quantification of the living metabolically active cells. In this period, MTT is reduced to purple formazan in the mitochondria of living cells. Formazan crystals in the wells were solubilized in 200 μL dimethyl sulfoxide (Sigma). Sample absorbance was measured at 570 nm wavelength using a microplate reader Synergy HT (BioTek Instruments Inc., Winooski, VT, USA). The viability was calculated considering controls containing serum-free medium (non-treated cells) as 100% viable. Results were obtained from three independent experiments (*n* = 3).

### 2.6. Determination of Anti-Inflammatory Activity of Olive-Leaf Extracts on Infected AGS Gastric Cells

Around 5 × 10^4^ cells/well of human gastric cells were used to seed 24-well plates (Sarstedt). Cells were incubated in cell culture medium at 37 °C under 5% CO_2_ in a humidifier incubator until monolayer formation. Olive-leaf extracts (E1 and E2) (1 mg/mL) were incubated with the cells at 37 °C under 5% CO_2_ for 2 h. Cells were washed twice with PBS and infected with 0.5 mL/well of *H. pylori* inoculum in serum-antibiotics-free medium (∼1 × 10^8^ CFU/mL for all tested strains). The cells and bacteria were incubated at 37 °C under 5% CO_2_ for 24 h to allow the bacteria to adhere to the gastric cells. Experimental control was formed by uninfected cells. Cell supernatants were collected and centrifuged at 12,000 rpm for 10 min at the end of incubation. Samples were stored at −20 °C until analysis was performed. ELISA assays were used to determine the amounts of secreted IL-8 cytokine in the supernatant of gastric cell samples. ELISA kit (Diaclone, Besançon, France), for the quantitation of IL-8, was used as described in the manufacturer’s instructions. The absorbance was measured at 450 nm using a microplate reader Synergy HT (BioTek Instruments Inc.). Such as in the absence of bacteria, gastric cells release small amounts of IL-8 [39]; titers of cytokine released by AGS cells were determined experimentally. Results were expressed as IL-8 production (pg/mL) of three independent experiments (*n* = 3).

### 2.7. Determination of Antioxidant Activity of Olive-Leaf Extracts against Intracellular Reactive Oxygen Species (ROS) Production on Infected AGS Gastric Cells

Oxidative stress induced by *H. pylori* infection was determined using AGS cells. Intracellular ROS were measured by the DCFH-DA (carboxy-2′,7′-dichloro-dihydrofluorescein diacetate) (Sigma) assay as described in Silvan et al. [40]. Cells were seeded (5 × 10^4^ cells per well) in 24-well plates and grown until 70% of confluence. Olive-leaf extracts (E1 and E2) (1 mg/mL) dissolved in serum-free medium were incubated with the cells at 37 °C under 5% CO_2_ for 2 h. Then, cells were washed with PBS and incubated with 20 μM DCFH-DA at 37 °C under 5% CO_2_ for 30 min. Cells were washed twice with PBS to remove the unabsorbed probe and infected with *H. pylori* strains suspended in serum-antibiotics-free medium (~1 × 10^8^ CFU/mL). ROS production was measured for 180 min in a fluorescent microplate reader Synergy HT (BioTek Instruments Inc.) using a λ_ex_ 485 nm and λ_em_ 530 nm. After being oxidized by intracellular oxidants, DCFH-DA changes to dichlorofluorescein (DCF) and emits fluorescence. Infected cells (non-treated) were used as oxidative control (100% of intracellular ROS production). Analysis was carried out in triplicate (*n* = 3). Results were expressed as % inhibition of ROS production.

### 2.8. Determination of Antibacterial Activity of Olive-Leaf Extracts against H. pylori Strains

Antibacterial activity of olive-leaf extracts (E1 and E2) against *H. pylori* was evaluated according to the protocol described by Silvan et al. [41]. Briefly, 1 mL of extracts (at 2 mg/mL final concentration) was added into flasks containing 4 mL of BB supplemented with 10% HS. Bacterial inoculum (50 μL of~1 × 10^8^ CFU/mL) was then added to the flasks under aseptic conditions. Cultures were incubated under stirring (150 rpm) in a VAIN at 37 °C for 24 h. Controls were prepared using sterile water instead of extracts. Serial decimal dilutions of cultures were prepared in saline solution (0.9% NaCl) after incubation. They were plated (20 μL) onto fresh MHB agar and incubated in a VAIN at 37 °C for 72 h. The number of CFU was assessed after incubation. The results of antibacterial activity were expressed as log CFU/mL (*n* = 3).

### 2.9. Verification of Bioactive Properties Using Pure Reference HT and OLE

Anti-inflammatory, antioxidant, and antibacterial properties of the major phenolic compounds in each extract (HT in extract E1 and OLE in extract E2) were investigated using commercial pure reference substances (Section 2.1) following the procedures described in the sections above. All analyses were carried out using the equivalent final concentration of these compounds in each extract (0.2 mg/mL HT in extract E1; 0.4 mg/mL OLE in extract E2).

### 2.10. Statistical Analysis

The results were reported as means ± standard deviations (SD) performed at least in triplicate (*n* = 3). Statistical analyses of the concentration of each quantified compound in both olive-leaf extracts were performed by *t*-test. Significant differences in the anti-inflammatory and antibacterial results were estimated by applying analysis of variance (ANOVA). Tukey’s least significant differences (LSD) test was used to evaluate the significance of these values. Statistical analysis of results for antioxidant activity was performed by ANOVA to compare values between strains treated with the same extract; and by *t*-test to compare values between both extracts for each strain. In all cases, differences were considered significant at *p* < 0.05. All statistical tests were performed with IBM SPSS Statistics for Windows, Version 26.0 (IBM Corp., Armonk, New York, NY, USA).

## 3. Results

### 3.1. Chemical Characterization of Olive-Leaf Extracts

HPLC-PAD-MS analysis showed that olive-leaf extracts (E1 and E2) were composed of a wide range of secondary metabolites, comprising mainly secoiridoids, phenolic compounds, and their derivatives, such as hydroxybenzoic and hydroxycinnamic acids, phenylethanols, secoiridoids, secoiridoid phenylethanols, cynnamoyl phenylethanols, flavones, flavonols, and flavanones (Table 1). This analysis allowed identification and quantification of twenty-five phenolic and secoiridoid compounds. Twenty-one of them (3,4-DHBA, *trans*-3,4-DHCA, *trans*-4-HCA, *trans*-3-M,4-HCA, *trans*-4,5-DCQA, 3,4-DHPE, 4-HPE, EA, EA 2-glucoside, EMA 2-glucoside, 3,4-DHPE-EA glucoside, 3,4-DHPE caffeoyl glucoside, apigenin 6,8-di-C-glucoside, apigenin 7-*O*-glucuronide, luteolin, luteolin 3′,7-di-*O*-glucoside, luteolin 7-*O*-glucoside, luteolin 4′-methyl ether 7-*O*-glucoside, quercetin, quercetin 3-*O*-glucoside, and eriodictyol 7-*O*-rutinoside) were identified unambiguously and four (3,4-DHBA and 3,4-DHPE glucosides) were identified tentatively.

From a quantitative point of view, these results showed that both extracts had substantial contents of phenolic and secoiridoid compounds, and extract E2 had considerably higher amounts of these compounds (29,155 mg/100 g) than the extract E1 (19,279 mg/100 g). Moreover, there were important qualitative differences between them: extract E1 contained highly hydrophilic compounds, among which HT, elenolic acid, and their glucosides, and several hydroxycinnamic acids were at higher concentrations (14,978, 2164, and 465 mg/100 g, respectively); and extract E2 contained moderately hydrophilic compounds, with the highest contents corresponding to OLE and verbascoside (20,471 and 6872 mg/100 g, respectively); and *trans*-4,5-DCQA, luteolin, flavonols, eriodictyol 7-*O*-rutinoside and eriodictyol 7-*O*-glucoside were absent in extract E1, whereas hydroxybenzoic and hydroxycinnamic acids, phenylethanols, and secoiridoids were absent or present at relatively low amounts in extract E2. Only most of the identified flavones were present at relatively equitative amounts in both olive-leaf extracts.

### 3.2. Anti-Inflammatory Activity of Olive-Leaf Extracts

Previously, we evaluated in vitro the secretion of different proinflammatory cytokines produced in *H. pylori*-infected AGS cells, with IL-8 being the most secreted cytokine (data not shown). For this reason, we selected IL-8 cytokine as a biomarker to evaluate the anti-inflammatory effect of olive-leaf extracts (E1 and E2) on AGS cells infected by different *H. pylori* strains. As shown in Table 2, AGS cells secreted basal levels of IL-8 (225.0 ± 21.2 pg/mL) and when AGS cells were infected with *H. pylori* strains, the IL-8 production was highly induced in a strain-dependent manner. All *H. pylori* strains significantly (*p* < 0.05) stimulated the IL-8 secretion by AGS cells with respect to uninfected cells (control). Hp48, Hp59, and Hp53, in this order, were the most proinflammatory strains, stimulating more than ten-fold the production of IL-8 with respect to uninfected AGS cells. Both extracts (E1 and E2), although in different proportions, significantly (*p* < 0.05) reduced IL-8 production by AGS-infected cells in all cases when compared with non-pretreated infected cells. Extract E2 was more effective, reducing IL-8 secretion between 71.5 to 94.9%, while extract E1 reduced IL-8 in a range from 30.6 to 74.4%. In addition, extract E2 reached the highest inhibition levels for the three most proinflammatory strains (Hp48, Hp59, and Hp53), with 94.9, 93.4, and 90.7%, respectively. Although both extracts were able to modulate IL-8 production on AGS cells infected by *H. pylori* strains, extract E2 was more effective. In this extract, OLE was the major phenolic compound (20,471 mg/100 g dw) (Table 1). However, when using a pure OLE standard at the concentration that was found in the extract E2 (0.4 mg/mL), no anti-inflammatory activity was observed against any of the *H. pylori* strains (data not shown).

### 3.3. Antioxidant Activity of Olive-Leaf Extracts against Intracellular Reactive Oxygen Species (ROS) Production

Antioxidant activity of olive-leaf extracts (E1 and E2) was evaluated against intracellular ROS produced by *H. pylori*-infected AGS cells. As shown in Table 3, extracts E1 and E2 inhibited ROS production in a similar way. Despite no significant differences (*p* > 0.05), similarities were obtained between olive leaf extract treatments for most of the strains, except for Hp48. Extract E2 reduced ROS production between 22.4 to 33.9%, while intracellular ROS reduction due to treatment with extract E1 ranged between 16.8 to 25.9%. No significant differences (*p* > 0.05) were observed between strains for the same extract treatment. As we described above, we evaluated, using pure reference substances, the role of HT and OLE on the inhibition of ROS production. Results obtained (data not shown) showed that they have no impact on ROS production at the concentrations at which they were found in extracts E1 and E2.

### 3.4. Antibacterial Activity of Olive-Leaf Extracts

Olive-leaf extracts (E1 and E2) were screened for their antibacterial activity against *H. pylori* strains, and the results obtained are presented in Table 4. Both extracts showed antibacterial activity, but in different degrees. Extract E1 showed bactericidal activity against all strains tested, indicating a relevant antibacterial activity. In contrast, antibacterial effect of extract E2 was strain-dependent, and it was bactericidal for strains Hp44 and Hp58. For Hp48 and Hp59, extract E2 reduced bacterial viability around 2 log of CFU/mL. In the case of Hp53, viability reduction was lower than 1 log of CFU/mL, while no significant differences were obtained for Hp61 with respect to control growth.

In our experiments, E1 was the extract with the highest antibacterial activity, and by far the most abundant phenolic compound was HT (13,743 mg/100 g dw) (Table 1). In the experiments we performed using a pure HT reference substance at the real concentration at which it was present in extract E1 (0.2 mg/mL), no antibacterial activity was observed against any of the *H. pylori* strains (data not shown).

## 4. Discussion

Due to the high correlation between *H. pylori* infection and gastric cancer, most therapeutic guidelines aim to eradicate this pathogen using a combination of antibiotics with a proton pump inhibitor in a triple or quadruple therapy [42]. However, there are currently two main issues that raise concerns about whether this therapeutic practice is the only possible option: first, the alarming global increase in antibiotic resistance, which has also affected the efficacy of treatments against *H. pylori* [43], and which can also lead to a significant disturbance in the microbiota [44]; and second, the relationship that has been observed between the eradication of *H. pylori* and the onset or worsening of other pathologies, such as esophageal reflux [45]. This situation has led to the increasing acceptance of alternative treatments to antibiotics, which can contribute to restoring the microbiota equilibrium, although they do not have the eradicating power of antibiotics. In this study, we have focused on evaluating the impact of two olive-leaf extracts (E1 and E2) on three key points closely related to the pathological progression of human *H. pylori* infection: inflammation, intracellular ROS production, and bacterial viability. The main difference between the two extracts is that extract E1 was richer in highly hydrophilic compounds, with HT representing the major component (13,743 mg/g) (Table 1). On the other hand, extract E2 was richer in moderately hydrophilic compounds, with OLE being the major compound (20,471 mg/g). The effectiveness of both extracts in modulating the inflammatory response and intracellular ROS production elicited by *H. pylori* in gastric cells was highly relevant, regardless of the strain of *H. pylori* causing the infection.

Some observations about the anti-inflammatory effect of phenolic compounds in extracts obtained from olive leaves and extra virgin olive oil on gastric epithelial cells has been previously described, but regarding inducing the cell inflammation by chemical agents. In this regard, for example, it has been previously confirmed that olive-leaf extract treatment in acidified ethanol-induced gastric ulcer in a rat model can prevent gastric inflammation by attenuating proinflammatory factors secretion, such as interleukin-1β (IL-1β) or tumor necrosis factor-α (TNF-α) [46]. There is also some clinical evidence of this effect. For instance, olive-leaf extract has previously demonstrated positive effects in clinical trials by modulating blood markers associated with inflammation and oxidative stress in healthy subjects and in patients affected by cardiovascular disease risk [47,48]. However, this work constitutes the first evidence of the direct anti-inflammatory and antioxidant effectiveness of olive-leaf extract in human gastric cells infected with different strains of *H. pylori* capable of stimulating an inflammatory response in these cells.

Despite the fact that the effectiveness of the extract E1 is not questioned, it appears that the moderately hydrophilic compounds in extract E2 have a greater impact on the anti-inflammatory activity. It has been previously described that HT and OLE, the main phenolic compounds in extract E1 and E2, respectively, showed a potent anti-inflammatory and antioxidant effect by inhibition of neutrophil degranulation, suppression of production, and release of inflammatory agents, and by inhibition of ROS production [17,49]. However, tests performed with pure reference substances of both compounds, as part of this study, failed to demonstrate that they are the only ones responsible for the observed effect. It may be possible that the whole effect of each extract involves the individual contribution of different compounds either additively or synergistically. Others have previously described, for example, that the high antioxidant capacity observed in olive-leaf extracts has been attributed to the synergic effect between the phenolic compounds present in comparison with the same compounds studied separately [50].

Regarding the antibacterial activity, the results indicated a greater strength of the highly hydrophilic compounds that constitute the extract E1. However, this effect showed no relationship with HT, the major compound of this extract. The antibacterial activity of HT is another controversial topic, since while some studies highlight its potent in vitro antibacterial activity even at very low concentrations [51], others focus on its limited antibacterial activity [52]. Other pure phenolic compounds that have been associated with the antibacterial activity of olive-leaf extracts, such as OLE and oleocanthal, were found at a very low concentration in extract E1 or were not detected, respectively. These results suggest that the antibacterial effect of olive-leaf extracts against *H. pylori* is, as we described above for antioxidant activity, probably due to the combined action or synergistic activity of different compounds. Some authors have previously reported strong antibacterial activity in vitro for olive-leaf extracts and olive oil against *H. pylori*. Sudjana et al. observed that although the extract used in their work did not have a broad spectrum of antimicrobial activity, it was particularly active against *H. pylori*, suggesting that olive-leaf extract may have a role in modulating the composition of the gastric microbiota due to its relevant antibacterial effect against *H. pylori* [20]. Similar observations were made by Romero et al. using virgin olive oil [32]. They incubated olive oil with a simulated gastric juice, observing that the phenolic compounds that diffused from the oil into the gastric juice had strong antibacterial activity against *H. pylori*. Among these compounds, the authors identified the dialdehydic form of decarboxymethyl ligstroside aglycon (oleocanthal) as the most active compound against *H. pylori*. However, these same authors obtained contradictory results in two subsequent clinical trials, and it was not possible to confirm in vivo the effectiveness observed in the experiments carried out in vitro. Among other factors, these discrepancies in the results were attributed to the variation in the phenolic composition of the olive oils used [33]. Further studies are required in order to understand these interactions and to identify the critical points whereby the effectiveness of these antibacterial compounds in the gastric mucosal environment is not exerted with the same potential as observed in vitro experiments.

Nevertheless, it is essential to test the in vivo response in clinical trials using appropriate markers that allow us to determine not only the antibacterial activity of these extracts against *H. pylori*, but also their impact as modulators of inflammation process and oxidative damage. Finally, this work shows the need to standardize the composition of olive-leaf extracts depending on the bioactivity required. Accordingly, hydrophilic, or less hydrophilic, compounds may be involved. This could be one of the main causes of some of the contradictory results observed using apparently similar extracts [32,33]. Therefore, it is imperative to have identified compounds that could serve as markers of extract activity and could be used as a chromatographic fingerprint to ensure homogeneity in the composition of the extracts with better bioactive response, regardless of the intrinsic variations between raw materials.

## 5. Conclusions

*H. pylori* infection may induce a chronic inflammatory response of gastric epithelial cells with increased oxidative damage mediated by ROS. In the present work, two different olive-leaf extracts (E1 and E2) were able to significantly decrease the inflammation response by reduction of IL-8 secretion, and to reduce the oxidative stress by inhibition of ROS production, in human gastric cells infected with different *H. pylori* strains. However, the effectiveness of each extract was determined by its composition. These results demonstrate the importance to standardize the composition of olive-leaf extracts according to the bioactive properties to be boosted. With a standardized preparation, olive-leaf extracts can be a tool of great potential in modulating the pathogenic effects produced by *H. pylori* infection. This possible application can also contribute to the development of new strategies for the valorization of olive by-products in order to achieve a sustainable use of these residues.

## Figures and Tables

**Table 1:** Ultraviolet absorption, mass spectrometric data, and quantification of main phenolic and secoiridoid compounds present in olive-leaf extracts (E1 and E2). Quantification is expressed as mean value ± standard deviation on dry matter (mg/100 g).

Compounds	Abs_max_ (nm)	[M + H]^+^ (*m*/*z*)	[M − H]^−^ (*m*/*z*)	Product Ions (-) (*m*/*z*)	Extract E1 (mg/100 g)	Extract E2 (mg/100 g)
***Hydroxybenzoic acids and glycosides***						
3,4-DHBA (Protocatechuic acid)	260/294		153.0	123.1, 109.0	7.9 ± 0.2	ND
3,4-DHBA glucoside	253/293		314.9	153.0, 136.9	6.4 ± 0.7	ND
Σ Hydroxybenzoic acids and glycosides					**14.3**	**ND**
***Hydroxycinnamic acids and derivatives***						
*trans*-3,4-DHCA (*trans*-caffeic acid)	234/296sh/322	181.0	179.0	135.1	140.0 ± 4.0 *	4.5 ± 0.1 *
*trans*-4-HCA (*trans*-4-coumaric acid)	233/296sh/308		163.0	145.0, 123.1, 119.1	209.0 ± 45.0 *	1.2 ± 0.1 *
*trans*-3-M,4-HCA (*trans*-ferulic acid)	236/295sh/322		193.1	178.1, 149.0	116.0 ± 4.0 *	5.0 ± 0.5 *
*trans*-4,5-DCQA (*trans*-4,5-dicaffeoylquinic acid)	235/300sh/326	517.1			ND	16.5 ± 0.2
Σ Hydroxycinnamic acids and derivatives					**465.0**	**27.2**
***Phenylethanols and glycosides***						
3,4-DHPG	232/278		169.0		20.1 ± 0.4 *	9.4 ± 0.5 *
3,4-DHPE (Hydroxytyrosol) + 3,4-DHPE glucoside 1	234/278		153.1 315.1	305.2 ^†^, 123.2 153.1, 123.2	13,743 ± 1659 *	182.0 ± 4.0 *
3,4-DHPE glucoside 2 + 3	230/278		315.0	153.1	965.0 ± 13.0 *	123.0 ± 1.0 *
4-HPE (Tyrosol)	232/275	161.2 [M+Na]^+^ 139.0			250.0 ± 6.0 *	9.1 ± 0.1 *
Σ Phenylethanols and glycosides					**14,978**	**323.5**
***Secoiridoids***						
EA (Elenolic acid)	239		241.1	165.1, 139.1, 127.1, 121.1, 111.2, 101.0	155.0 ± 14.0	ND
EA 2-glucoside (Oleoside 11-methyl ester)	238		403.1	807.2 ^†^, 223.1, 179.0, 119.1, 112.9	1352 ± 49.0 *	84.4 ± 4.6 *
EMA 2-glucoside (Secoxyloganin)	237		403.0	807.2 ^†^, 223.1, 179.1, 121.1, 119.2, 113.1	657.0 ± 158.0	ND
Σ Secoiridoids					**2164**	**84.4**
***Secoiridoid phenylethanols***						
3,4-DHPE-EA glucoside (Oleuropein)	234/280	563.2 [M+Na]^+^	539.2		355.0 ± 57.0 *	20,471 ± 1061*
4-HPE-EA-glucoside (Ligustroside)	234/280		523.0		99.3 ± 9.3 *	360 ± 16 *
Σ Secoiridoid phenylethanols					**454.3**	**20,831**
***Cynnamoyl phenylethanol glycosides***						
3,4-DHPE caffeoyl glucoside (Verbascoside)	234/285sh/330	647.2 [M+Na]^+^	623.2		161.0 ± 11.0 *	6872 ± 230 *
Σ Cynnamoyl phenylethanol glycosides					**161.0**	**6872**
***Flavones***						
Apigenin 6,8-di-C-glucoside	254/265sh/348	595.2			39.3 ± 1.4 *	24.2 ± 0.2 *
Apigenin 7-*O*-rutinoside (Isorhoifolin)	266/341	579.1	576.8		109 ± 1 *	122 ± 5 *
Apigenin 7-*O*-glucuronide	266/338		445.1		76.4 ± 7.1	64 ± 6.0
Luteolin	253/265/348	287.0	285.1	284.1	ND	17.1 ± 1.2
Luteolin 3′,7-di-*O*-glucoside	267/342		609.1		39.9 ± 1.4 *	69.6 ± 2.3 *
Luteolin 7-*O*-glucoside	254/268sh/348	449.1	447.1	895.0 ^†^	655.0 ± 22.0 *	513.0 ± 46.0 *
Luteolin 4′-methyl ether 7-*O*-glucoside (Diosmin)	252/266/347	609.2	606.9	461.0, 299.1	123.0 ± 11.0	111.0 ± 12.0
Σ Flavones					**1042.6**	**920.9**
***Flavonols***						
Quercetin 3-*O*-glucoside (Isoquercitrin)	233/281/322	465.1	463.1		ND	9.1 ± 0.6
Quercetin 3-rhamnoside (Quercitrin)	253/295sh/342	449.1	447.1		ND	10.8 ± 0.2
Quercetin	254/300sh/370	303.0			ND	31.4 ± 0.5
Σ Flavonols					**ND**	**51.3**
***Flavanones***						
Eriodictyol 7-*O*-rutinoside	233/283/328		594.6	449.1, 286.9, 151.3	ND	22.3 ± 3.1
Eriodictyol 7-*O*-glucoside	232/277/340	451.1	449.1		ND	22.8 ± 1.0
Σ Flavanones					**ND**	**45.1**
Total phenolic and secoiridoid compounds					**19,051**	**28,630**

ND: not detected; sh: peak shoulder; DHBA: dihydroxybenzoic acid; DHCA: dihydroxycinnamic acid; HCA: hydroxycinnamic acid; 3-M,4-HCA: 3-Methoxy-4-hydroxycinnamic acid; DCQA: dicaffeoylquinic acid; DHPG: 3,4-dihydroxyphenylglycol; DHPE: dihydroxyphenylethanol; HPE: hydroxyphenylethanol; EA: Elenolic acid; EMA 2-glucoside: EA monoaldehyde isomer 2-glucoside. ^†^ Double-charged ion [M-2H]^2−^. * Data marked with asterisk in the same row indicate significant difference between values (*p* ≤ 0.05).

**Table 2:** Effect of olive-leaf extracts (E1 and E2) on IL-8 production in AGS cells infected by *H. pylori* strains. Values of IL-8 production are expressed as pg/mL (mean ± standard deviation) (*n* = 3).

Strains	Control (Cells without Extracts)	Extract E1	Extract E2
Uninfected AGS cells	225.0 ± 21.2 ^A-a^	215.0 ± 10.2 ^a^	205.0 ± 16.2 ^a^
Hp44	1119.4 ± 112.3 ^C-b^	318.8 ± 23.0 ^a^ (71.5%) *	212.5 ± 5.3 ^a^ (81.0%) *
Hp48	3116.3 ± 49.5 ^F-c^	2163.0 ± 139.7 ^b^ (30.6%) *	158.1 ± 6.2 ^a^ (94.9%) *
Hp53	2413.8 ± 30.1 ^D-c^	618.1 ± 69.8 ^b^ (74.4%) *	225.0 ± 76.0 ^a^ (90.7%) *
Hp58	733.1 ± 6.2 ^B-c^	421.9 ± 39.8 ^b^ (42.5%) *	166.3 ± 5.3 ^a^ (77.3%) *
Hp59	2825.6 ± 94.6 ^E-c^	1911.9 ± 9.7 ^b^ (32.3%) *	187.5 ± 24.7 ^a^ (93.4%) *
Hp61	955.0 ± 24.7 ^B,C-b^	374.4 ± 110.5 ^a^ (60.8%) *	271.9 ± 8.0 ^a^ (71.5%) *

^a–c^ Values in the same row marked with different lowercase letters indicate significant differences by ANOVA post hoc LSD Tukey test (*p* ≤ 0.05). ^A–F^ Values in the control column marked with different uppercase letters indicate significant differences by ANOVA post hoc LSD Tukey test (*p* ≤ 0.05). * Values showed in brackets indicate the % inhibition of IL-8 production respect to non-pretreated infected cells (control).

**Table 3:** Protective effect of olive-leaf extracts (E1 and E2) on intracellular ROS production by *H. pylori*-infected AGS cells. Values are expressed as % inhibition of ROS production (mean ± standard deviation) (*n* = 3).

Strain	Extract E1	Extract E2
Hp44	18.11 ± 3.31^a^	22.41 ± 5.54 ^a^
Hp48	16.79 ± 1.71 ^a,^*	29.87 ± 3.93 ^a,^*
Hp53	25.93 ± 4.81 ^a^	33.88 ± 2.37 ^a^
Hp58	23.05 ± 2.10 ^a^	29.99 ± 2.07 ^a^
Hp59	22.17 ± 3.98 ^a^	25.73 ± 1.86 ^a^
Hp61	25.92 ± 2.25 ^a^	30.59 ± 5.06 ^a^

^a^ Values in the same column marked with different lowercase letters indicate significant differences by ANOVA post hoc LSD Tukey test (*p* ≤ 0.05). * Values in the same row marked with asterisks indicate significant differences between the treatments with extracts for each strain determined by *t*-test (*p* < 0.05).

**Table 4:** Antibacterial activity of olive-leaf extracts (at 2 mg/mL) on the viable counts of different *H. pylori* strains after 24 h of treatment. Results are expressed as log CFU/mL ± standard deviation (*n* = 3).

Strains	Control Growth	Extract E1		Extract E2	
		log CFU/mL	log Reduction	log CFU/mL	log Reduction
Hp44	7.82 ± 0.03 ^b^	<1.5 *^,a^	7.82	<1.5 *^,a^	7.82
Hp48	7.69 ± 0.02 ^c^	<1.5 *^,a^	7.69	5.55 ± 0.13 ^b^	2.14
Hp53	7.48 ± 0.02 ^c^	<1.5 *^,a^	7.48	6.83 ± 0.01 ^b^	0.65
Hp58	6.69 ± 0.33 ^b^	<1.5 *^,a^	6.69	<1.5 *^,a^	6.69
Hp59	7.74 ± 0.01 ^c^	<1.5 *^,a^	7.74	5.95 ± 0.03 ^b^	1.79
Hp61	7.56 ± 0.03 ^b^	<1.5 *^,a^	7.56	7.51 ± 0.03 ^b^	0.05

* Colony forming unit, detection limit was 1.5 log CFU/mL (30 CFU per plate). ^a–c^ Log CFU/mL values in the same row marked with different letters indicate significant differences by ANOVA post hoc LSD Tukey test (*p* ≤ 0.05).

## Data Availability

The data presented in this study are available in this manuscript.

## References

[1] Gullón P., Gullón B., Astray G., Carpena M., Fraga-Corral M., Prieto M.A., Simal-Gandara J. (2020). Valorization of by-products from olive oil industry and added-value applications for innovative functional foods. Food Res. Int..

[2] Kiritsakis K., Goula A.M., Adamopoulos K.G., Gerasopoulos D. (2018). Valorization of olive leaves: Spray drying of olive leaf extract. Waste Biomass Valor..

[3] Flamminii F., Di Mattia C.D., Difonzo G., Neri L., Faieta M., Caponio F., Pittia P. (2019). From by-product to food ingredient: Evaluation of compositional and technological properties of olive-leaf phenolic extracts. J. Sci. Food Agric..

[4] Romero-García J.M., Niño L., Martínez-Patiño C., Álvarez C., Castro E., Negro M.J. (2014). Biorefinery based on olive biomass. State of the art and future trends. Bioresour. Technol..

[5] El S.N., Karakaya S. (2009). Olive tree (*Olea europaea*) leaves: Potential beneficial effects on human health. Nutr. Rev..

[6] Espeso J., Isaza A., Lee J.Y., Sörensen P.M., Jurado P., Avena-Bustillos R.J., Olaizola M., Arboleya J.C. (2021). Olive leaf waste management. Front. Sustain. Food Syst..

[7] Benavente-García O., Castillo J., Lorente J., Ortuño A., Del Rio J.A. (2000). Antioxidant activity of phenolics extracted from *Olea europaea* L. leaves. Food Chem..

[8] Özcan M.M., Matthäus B. (2017). A review: Benefit and bioactive properties of olive (*Olea europaea* L.) leaves. Eur. Food Res. Technol..

[9] Qabaha K., Al-Rimawi F., Qasem A., Naser S.A. (2018). Oleuropein is responsible for the major anti-inflammatory effects of olive leaf extract. J. Med. Food..

[10] Andrikopoulos N.K., Kaliora A.C., Assimopoulou A.N., Papageorgiou V.P. (2002). Inhibitory activity of minor polyphenolic and non-polyphenolic constituents of olive oil against *in-vitro* low-density lipoprotein oxidation. J. Med. Food.

[11] Visioli F., Poli A., Gall C. (2002). Antioxidant and other biological activities of phenols from olives and olive oil. Med. Res. Rev..

[12] Wang L., Geng C., Jiang L., Gong D., Liu D., Yoshimura H., Zhong L. (2008). The anti-atherosclerotic effect of olive leaf extract is related to suppressed inflammatory response in rabbits with experimental atherosclerosis. Eur. J. Nutr..

[13] Fakhraei N., Abdolghaffari A.H., Delfan B., Abbasi A., Rahimi N., Khansari A., Rahimian R., Dehpour A.R. (2014). Protective effect of hydroalcoholic olive leaf extract on experimental model of colitis in rat: Involvement of nitrergic and opioidergic systems. Phytother. Res..

[14] Vezza T., Algieri F., Rodríguez-Nogales A., Garrido-Mesa J., Utrilla M.P., Talhaoui N., Gómez-Caravaca A.M., Segura-Carretero A., Rodríguez-Cabezas M.E., Monteleone G. (2017). Immunomodulatory properties of *Olea europaea* leaf extract in intestinal inflammation. Mol. Nutr. Food Res..

[15] Fernández-Prior Á., Bermúdez-Oria A., Millán-Linares M.D.C., Fernández-Bolaños J., Espejo-Calvo J.A., Rodríguez-Gutiérrez G. (2021). Anti-inflammatory and antioxidant activity of hydroxytyrosol and 3,4-dihydroxyphenyglycol purified from table olive effluents. Foods.

[16] Karković Marković A., Torić J., Barbarić M., Jakobušić Brala C. (2019). Hydroxytyrosol, tyrosol and derivatives and their potential effects on human health. Molecules.

[17] Bedouhene S., Moulti-Mati F., Dang P.M., El-Benna J. (2017). Oleuropein and hydroxytyrosol inhibit the N-formyl-methionyl-leucyl-phenylalanine-induced neutrophil degranulation and chemotaxis via AKT, p38, and ERK1/2 MAP-Kinase inhibition. Inflammopharmacology.

[18] Bulotta S., Oliverio M., Russo D., Procopio A., Ramawat K., Mérillon J.M. (2013). Biological activity of oleuropein and its derivatives. Natural Products.

[19] Gullón B., Gullón P., Eibes G., Cara C., De Torres A., López-Linares J.C., Ruiz E., Castro E. (2018). Valorisation of olive agro-industrial by-products as a source of bioactive compounds. Sci. Total Environ..

[20] Sudjana A.N., D’Orazio C., Ryan V., Rasool N., Ng J., Islam N., Riley T.V., Hammer K.A. (2009). Antimicrobial activity of commercial *Olea europaea* (olive) leaf extract. Int. J. Antimicrob. Agents.

[21] WHO, International Agency for Research on Cancer (1994). IARC Monographs on the Evaluation of Carcinogenic Risks to Humans, Volume 61: Schistosomes, Liver Flukes and Helicobacter pylori.

[22] Kusters J.G., van Vliet A.H.M., Kuipers E.J. (2006). Pathogenesis of *Helicobacter pylori* infection. Clin. Microbiol. Rev..

[23] Konturek P.C., Konturek S.J., Brzozowski T. (2009). *Helicobacter pylori* infection in gastric cancerogenesis. J. Physiol. Pharmacol..

[24] Denic M., Touati E., De Reuse H. (2020). Review: Pathogenesis of *Helicobacter pylori* infection. Helicobacter.

[25] White J.R., Winter J.A., Robinson K. (2015). Differential inflammatory response to *Helicobacter pylori* infection: Etiology and clinical outcomes. J. Inflamm. Res..

[26] Butcher L.D., den Hartog G., Ernst P.B., Crowe S.E. (2017). Oxidative stress resulting from *Helicobacter pylori* infection contributes to gastric carcinogenesis. Cell. Mol. Gastroenter. Hepatol..

[27] Kountouras J., Chatzopoulos D., Zavos C. (2001). Reactive oxygen metabolites and upper gastrointestinal diseases. Hepatogastroenterology.

[28] Mittal M., Siddiqui M.R., Tran K., Reddy S.P., Malik A.B. (2014). Reactive oxygen species in inflammation and tissue injury. Antioxid. Redox Signal..

[29] Wroblewski L.E., Peek R.M., Wilson K.T. (2010). *Helicobacter pylori* and gastric cancer: Factors that modulate disease risk. Clin. Microbiol. Rev..

[30] Correa P., Haenszel W., Cuello C. (1975). A model for gastric cancer epidemiology. Lancet.

[31] Alesci A., Miller A., Tardugno R., Pergolizzi S. (2021). Chemical analysis, biological and therapeutic activities of *Olea europaea* L. extracts. Nat. Prod. Res..

[32] Romero C., Medina E., Vargas J., Brenes M., De Castro A. (2007). In vitro activity of olive oil polyphenols against *Helicobacter pylori*. J. Agric. Food Chem..

[33] Castro M., Romero C., de Castro A., Vargas J., Medina E., Millán R., Brenes M. (2012). Assessment of *Helicobacter pylori* eradication by virgin olive oil. Helicobacter.

[34] Silvan J.M., Gutiérrez-Docio A., Moreno-Fernandez S., Alarcón-Cavero T., Prodanov M., Martinez-Rodriguez A.J. (2020). Procyanidin-rich extract from grape seeds as a putative tool against *Helicobacter pylori*. Foods.

[35] Medina E., Brenes M., Romero C., García A., de Castro A. (2007). Main antimicrobial compounds in table olives. J. Agric. Food Chem..

[36] Perestrelo R., Lu Y., Santos S.A.O., Silvestre A.J.D., Neto C.P., Câmara J.S., Rocha S.M. (2012). Phenolic profile of Sercial and Tinta Negra *Vitis vinifera* L. grape skins by HPLC–DAD–ESI-MS^n^: Novel phenolic compounds in *Vitis vinifera* L. grape. Food Chem..

[37] Michel T., Khlif I., Kanakis P., Termentzi A., Allouche N., Halabalaki M., Skaltsounis A.L. (2015). UHPLC-DAD-FLD and UHPLC-HRMS/MS based metabolic profiling and characterization of different *Olea europaea* organs of Koroneiki and Chetoui varieties. Phytochem. Let..

[38] Silvan J.M., Michalska-Ciechanowska A., Martinez-Rodriguez A.J. (2020). Modulation of antibacterial, antioxidant, and anti-inflammatory properties by drying of *Prunus domestica* L. plum juice extracts. Microorganisms.

[39] Silvan J.M., Mingo E., Martinez-Rodriguez A.J. (2017). Grape seed extract (GSE) modulates *Campylobacter* pro-inflammatory response in human intestinal epithelial cell lines. Food Agric. Immunol..

[40] Silvan J.M., Gutierrez-Docio A., Guerrero-Hurtado E., Domingo-Serrano L., Blanco-Suarez A., Prodanov M., Alarcon-Cavero T., Martinez-Rodriguez A.J. (2021). Pre-treatment with grape seed extract reduces inflammatory response and oxidative stress induced by *Helicobacter pylori* infection in human gastric epithelial cells. Antioxidants.

[41] Silvan J.M., Zorraquin-Peña I., Gonzalez de Llano D., Moreno-Arribas M.V., Martinez-Rodriguez A.J. (2018). Antibacterial activity of glutathione-stabilized silver nanoparticles against *Campylobacter* multidrug-resistant strains. Front. Microbiol..

[42] Guevara B., Cogdill A.G. (2020). *Helicobacter pylori*: A review of current diagnostic and management strategies. Dig. Dis. Sci..

[43] Alba C., Blanco A., Alarcón T. (2017). Antibiotic resistance in *Helicobacter pylori*. Curr. Opin. Infect. Dis..

[44] Chen C.C., Liou J.M., Lee Y.C., Hong T.C., El-Omar E.M., Wu M.S. (2021). The interplay between *Helicobacter pylori* and gastrointestinal microbiota. Gut Microbes.

[45] Zhao Y., Li Y., Hu J., Wang X., Ren M., Lu G., Lu X., Zhang D., He S. (2020). The effect of *Helicobacter pylori* eradication in patients with gastroesophageal reflux disease: A meta-analysis of randomized controlled studies. Dig. Dis..

[46] Al-Quraishy S., Othman M.S., Dkhil M.A., Abdel Moneim A.E. (2017). Olive (*Olea europaea*) leaf methanolic extract prevents HCl/ethanol-induced gastritis in rats by attenuating inflammation and augmenting antioxidant enzyme activities. Biomed. Pharmacother..

[47] Lockyer S., Corona G., Yaqoob P., Spencer J.P.E., Rowland I. (2015). Secoiridoids delivered as olive leaf extract induce acute improvements in human vascular function and reduction of an inflammatory cytokine: A randomised, double-blind, placebo-controlled, cross-over trial. Br. J. Nutr..

[48] Lockyer S., Rowland I., Spencer J.P.E., Yaqoob P., Stonehouse W. (2017). Impact of phenolic-rich olive leaf extract on blood pressure, plasma lipids and inflammatory markers: A randomised controlled trial. Eur. J. Nutr..

[49] Bucciantini M., Leri M., Nardiello P., Casamenti F., Stefani M. (2021). Olive polyphenols: Antioxidant and anti-inflammatory properties. Antioxidants.

[50] Vogel P., Kasper Machado I., Garavaglia J., Terezinha Zani V., Souza D., Bosco S.M.D. (2015). Polyphenols benefits of olive leaf (*Olea europaea* L.) to human health. Nutr. Hosp..

[51] Wei J., Wang S., Pei D., Qu L., Li Y., Chen J., Di D., Gao K. (2018). Antibacterial activity of hydroxytyrosol acetate from olive leaves (*Olea*
*europaea* L.). Nat. Prod. Res..

[52] Medina-Martínez M.S., Truchado P., Castro-Ibáñez I., Allende A. (2016). Antimicrobial activity of hydroxytyrosol: A current controversy. Biosci. Biotechnol. Biochem..

